# Label-Free Biological and Chemical Sensing Using Whispering Gallery Mode Optical Resonators: Past, Present, and Future

**DOI:** 10.3390/s17030540

**Published:** 2017-03-08

**Authors:** Judith Su

**Affiliations:** College of Optical Sciences, Department of Chemistry & Biochemistry, University of Arizona Cancer Center, University of Arizona, Tucson, AZ 85721, USA; judy@optics.arizona.edu

**Keywords:** label-free, biosensing, chemical sensing, optical resonator, single molecule, microcavity, whispering gallery mode

## Abstract

Sensitive and rapid label-free biological and chemical sensors are needed for a wide variety of applications including early disease diagnosis and prognosis, the monitoring of food and water quality, as well as the detection of bacteria and viruses for public health concerns and chemical threat sensing. Whispering gallery mode optical resonator based sensing is a rapidly developing field due to the high sensitivity and speed of these devices as well as their label-free nature. Here, we describe the history of whispering gallery mode optical resonator sensors, the principles behind detection, the latest developments in the fields of biological and chemical sensing, current challenges toward widespread adoption of these devices, and an outlook for the future. In addition, we evaluate the performance capabilities of these sensors across three key parameters: sensitivity, selectivity, and speed.

## 1. Introduction

The field of biological and chemical sensing using whispering gallery mode (WGM) optical resonators is rapidly expanding chiefly due to the high sensitivity, quick response time, and label-free nature of these devices. WGM resonators have inherently high sensitivity due to their long (tens of nanoseconds) photon confinement time and have a fast time (tens of seconds) response due to their compactness. In addition, WGM resonators belong to a class of biosensors that are label-free, meaning there is no need for a bio-recognition element such as a fluorescent or radioactive tag. The high sensitivity and speed of WGM resonators combined with the ability to detect molecules in their native state has great future potential for basic and applied research such as reliable single molecule detection, observing transient protein folding states, measuring protein secretions from a single cell, single molecule DNA sequencing, high-throughput drug screening, environmental monitoring, early disease diagnostics, monitoring vaccine efficacy, chemical threat sensing, and biodefense, among others. For many applications, in particular those impacting human health such as chemical threat sensing, biodefense, and medical diagnostics, the fast response time of WGM sensors is key. WGM sensors are among the most sensitive and fastest transducers in existence, capable in some cases of detecting attomolar concentrations of protein in under a minute [1].

**Figure 1:**
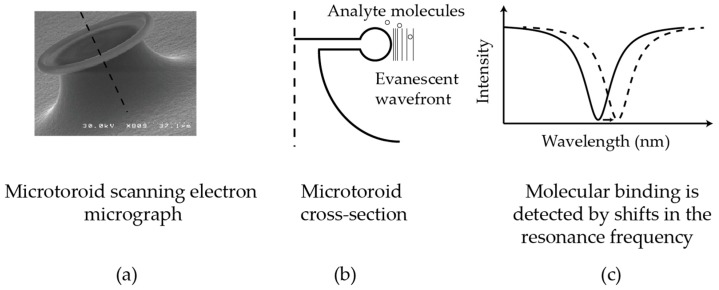
The microtoroid is an example of a whispering gallery mode optical resonator: (**a**) a scanning electron micrograph of a microtoroid; (**b**) a schematic of the evanescent wavefront interacting with molecules near the microtoroid (not to scale); and (**c**) molecules binding to the toroid’s surface changes the resonant frequency of the device. Whispering gallery mode resonators provide enhanced sensitivity as light interacts with the analyte molecules multiple times.

For certain geometries, WGM resonators may be thought of as a waveguide with the ends joined together. Thus, light at particular (resonant) frequencies circulates continuously. Because of the bent optical path, there is leakage of the light from the cavity’s surface into the surrounding medium (Figure 1). This leaked light allows bound analytes to be re-interrogated many times, providing orders of magnitude greater sensitivity than conventional label-free sensing techniques such as Biacore surface plasmon resonance [2] which require nanomolar concentrations of analyte to bind for a detectable signal to be observed. The high sensitivity of WGM resonators enable smaller sample volumes to be used, enabling experiments involving hard to obtain reagents as well as reducing cost.

As optical sensors, WGM resonators have the advantage of not being affected by electromagnetic interference and are capable of remote detection. WGM resonators are also refractive index sensors. These sensors measure changes in the index of refraction of the sensor as particles bind. In the case of WGM sensors, this is done by measuring corresponding changes in the resonance frequency of the cavity (Figure 1).

Other kinds of resonator-based optical sensors include Fabry–Perot resonators [3] and photonic crystal cavities [4]. Compared to Fabry–Perot resonators, WGM resonators have resonant modes with significantly higher quality factors (*Q*) and thus are more sensitive sensors. The *Q*-factor is a figure of merit used for all resonators and is proportional to how many times light circulates around the cavity. *Q* may be expressed as:

(1) $$Q=\;\frac{\omega_{0}}{\Delta \omega },$$

where $\omega_{0}$ is the center frequency of the resonant mode and Δω is the width the mode. From Equation (1) we can see that narrower mode widths correspond to higher *Q* values. Higher *Q* enables both more interrogation of a bound analyte and improved wavelength resolution, thus enabling smaller particles to be detected. In comparison to photonic crystal cavities, WGM resonators are easier to fabricate [5,6] and have a larger *Q*-factor to mode volume (*Q*/V) ratio (Purcell factor) [7], resulting in larger power densities (circulating intensities).

WGM sensors have other advantages as well. Compared to other nanoscale sensors, such as nanowires [8], cantilevers [9], and plasmonic particles [10], WGM sensors have a larger effective capture area, thus making analyte detection events more likely. In addition, WGM sensors operate well in water. In contrast, mechanical cantilevers, while having high sensitivity in air and vacuum [11], are dampened in water and typically do not operate well in aqueous environments. At the level of individual particles, WGM sensors have been used to detect and size bacteria [12], viruses [13,14], DNA [15], exosomes [16], ribosomes [1], antibodies [1], and single protein molecules [1,17,18].

## 2. History

**Figure 2:**
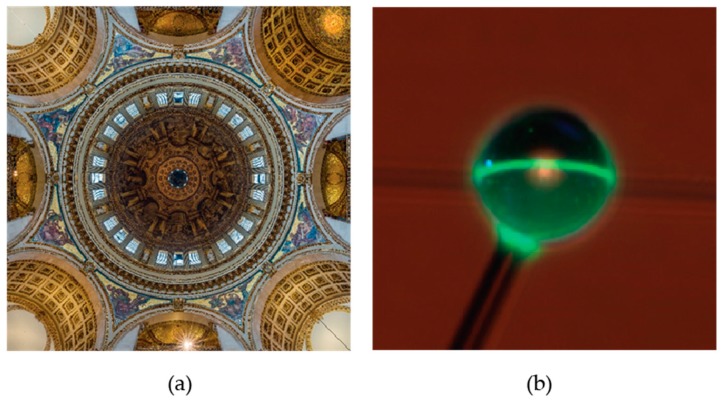
(**a**) A view from underneath the dome of St. Paul’s Cathedral in London. The gallery underneath the dome is an example of an acoustic whispering gallery. Whispering gallery mode optical resonators were named after the acoustic whispering galleries as follows a similar principle, but with electromagnetic instead of acoustic waves. Photo by David Iliff. License: CC BY-SA3.0. (**b**) Photograph of light orbiting within a microsphere optical resonator. The sphere has been doped with erbium and is approximately 70 µm in diameter. Light has been evanescently coupled into the microsphere using an optical fiber. Reprinted by permission from Macmillan Publishers Ltd.: [7], copyright (2003).

Whispering gallery mode resonators are named after the acoustic whispering gallery, which was described by Lord Rayleigh in 1910 [19,20,21]. Lord Rayleigh noticed that when he stood in the gallery under the dome of St. Paul’s Cathedral in London (Figure 2), whispers on one side of the gallery could be heard 40 m away, as sound would skirt along the edges of the gallery with negligible loss. Whispering gallery mode optical resonators employ the same principle, but with light instead of sound. Light is guided along the perimeter of these devices by multiple (near-) total internal reflections. At the resonance wavelength, light returns in-phase every revolution, causing constructive interference and an increase in the light stored in the cavity.

Whispering gallery mode optical resonators were predicted in the early 20th century [22], soon after Mie theory was developed, which provided a theoretical basis for the resonant modes within these cavities [23]. Evidence of such optical whispering modes was first observed in 1961 in 1–2 mm spheres of CaF_2_ which had been doped with samarium and placed in a dewar of liquid hydrogen [24]. In the 1980s, narrow light resonances were observed in liquid droplets [25,26,27,28] that had been irradiated with an incident laser beam. These resonances are called whispering gallery mode resonances or morphology-dependent resonances (MDR) [29] and are attributed to electromagnetic waves caused by multiple internal reflections within the cavity. They are an example of a surface tension induced microcavity (STIM) [30]. The *Q-*factor of the resonators in these cases was ~10^4^ [31]. These resonators, however, were not very stable as they were made of liquid and can undergo oscillations which cause them to deviate from perfect spherical shape.

Close to a decade later, robust, solid silica microsphere STIMs [31] (Figure 2b) were formed by melting the tip of an optical fiber using a torch. Soon afterward, microsphere resonators were shown to demonstrate resonant modes with quality (*Q*) factors ~10^10^ at 633 nm [32]. This is on the order of the largest experimentally observed *Q*-factors to date and was achieved by fire-polishing the surface of the sphere to reduce scattering losses.

The *Q*-values of WGM resonators are limited by scattering from surface imperfections, contamination, radiative losses, bending losses due to the curvature of the cavity, and material absorption [32]. According to Lorenz–Mie theory, *Q* can reach as high as 10^21^ in the absence of material absorption [33]. This limit comes from the leakage of light caused by the bent optical path. High sensitivity biochemical detection requires maximizing the cavity *Q*-factor, as narrower resonance linewidths enable smaller spectral shifts to be measured. We note that practical sensing applications can also be achieved with more modest *Q* values [34].

In 1993 mechanical and chemical sensing using WGM sensors was first proposed [35]. Around the same time in 1993 and later in 1994, fluorophore-containing liquid droplets were shown to be sensitive absorption indicators, capable of measuring changes within the droplet in the concentration of rhodamine dye. Internal changes in droplet pH were also measured by monitoring changes in the absorption of an indicator dye [36,37]. These droplets were produced by an aerosol generator which created very monodisperse droplets 20–40 microns in diameter depending on the settings. In the mid-1990s, the concept of using these devices as biosensors was proposed [38] and in 2002, these microsphere resonators were used to measure the adsorption of micromolar concentrations of bovine serum albumin (BSA) as well as the binding of streptavidin to biotinylated BSA [39]. In 2003, microtoroid optical cavities were invented [30] with the goal of integrating a high-*Q* device on a chip. This is a first step toward making multiplexed devices capable of performing many measurements in parallel. The decreased sensor surface area relative to the mode volume of the toroid which compared to the microsphere means that more of the sensor’s surface can be used for analyte detection events. A few years later, microspheres [13] and microtoroids [14] were shown able to detect single influenza A virus particles. Since this time, a wide variety of biological and chemical sensing experiments have been performed with WGM resonators. In a hybrid dual-resonator approach, microspheres have recently been coupled to 70 nm radius silica core gold nanoshells to boost signal in order to detect single BSA (~14 nm) molecules [18], however, this approach is limited in that there are only two hotspots per nanoparticle and one nanoparticle per microsphere limiting the active detection area of the sensor. Recently, frequency-locked microtoroid optical resonators were shown capable of detecting single macromolecules at attomolar concentrations [1]. As detection strength signal strength scales with particle volume, detecting smaller particles requires significant improvements in the signal-to-noise ratio of these devices.

## 3. Overview of Different Types Whispering Gallery Mode Optical Resonators

**Figure 3:**
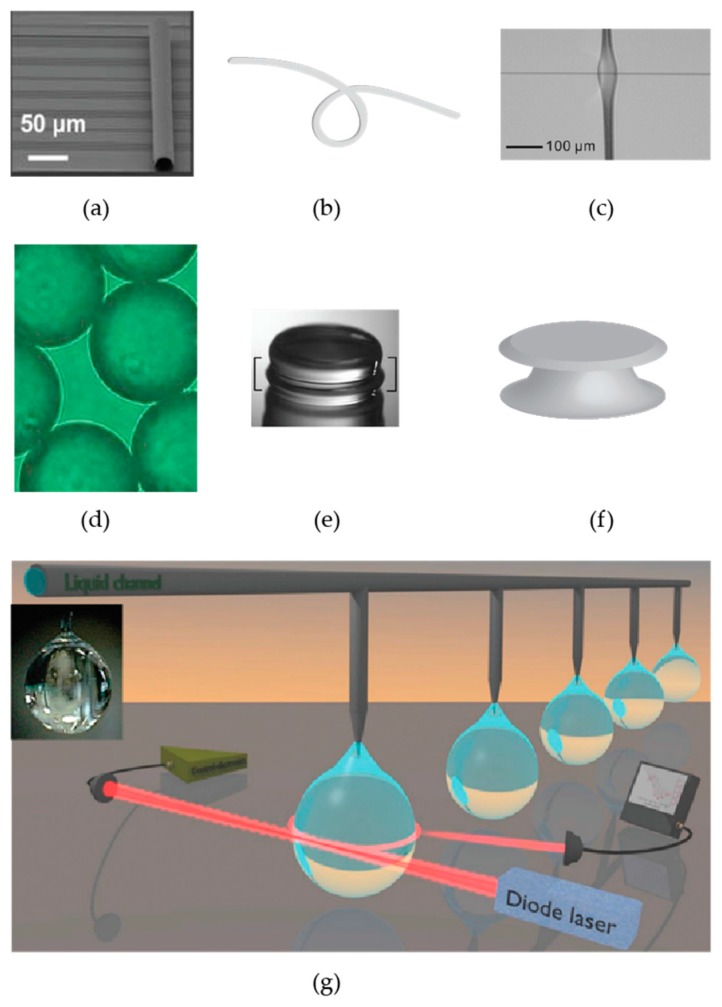
Some examples of different optical microcavities. (**a**) Vertical optical ring resonators. Adapted from [46]; (**b**) Knot resonators; (**c**) Microbubble resonators. Reprinted from [47], with the permission of AIP Publishing; (**d**) Polystyrene microsphere resonators. These resonators are an inexpensive way to perform many experiments in parallel. Reprinted from [48], with the permission of AIP Publishing; (**e**) Crystalline CaF_2_ cavities. The resonator is shown in the area between the white brackets and is ~5.5 mm in diameter. Reprinted from [54]; (**f**) Microdisk resonators; and (**g**) Liquid droplet resonators. The inset is of a liquid paraffin oil drop. Adapted from [44]. Copyright Wiley-VCH Verlag GmbH & Co. KGaA.

There are many different optical microcavities made of different materials and with different geometries (Figure 3). A partial list includes microrings [40,41], disks [12], knots/coils [42], bottles [43], droplets [44], goblets [45], rolled-up microcavities [46], bubbles [47], polystyrene microspheres [48,49], asymmetric cavities [50], cylinders [51], vertically coupled race track resonators [52], nearly spherical diamond resonators [53], crystalline CaF_2_ cavities [54], and liquid-core optical ring resonators (LCORR) [55]. White-light WGM resonators have also been developed [56]. These resonators have the advantage that a tunable laser is not needed because resonances can be excited using a white light source. With white-light WGM resonators, biochemical sensing experiments can be performed by measuring changes in the photon lifetime of the resonator using cavity ring-up spectroscopy [57].

The most commonly used resonators used for biological and chemical sensing are silica microrings [58], microtoroids [30], and micropheres [13]. Microrings [59,60] have been commercialized by Genalyte and can perform 32 assays simultaneously, similar to Biacore surface plasmon resonance. Although they can perform parallel assays, microring resonators have lower *Q* values than either a microsphere or toroid. This is because they retain lithographic blemishes caused during manufacturing and also light is lost via interaction with their substrate. A typical level of performance for microring resonators is picomolar detection of proteins [58].

Microrings are easily fabricated on chip and thus easily integratable with microfluidics, which facilitates surface functionalization due to improved liquid handling and smaller fluid volumes [61]. Functionalization of all surfaces may however lead to undesirable capturing of analyte away from the sensing surface, which could lead to reduced sensitivity at low concentrations. In the case of colloidal spheres, they can be functionalized before being deposited on a surface. For both microrings and colloidal spheres, multiplexing enables a separate resonator to be used as a control reference.

Different cavity shapes can also enable easier coupling, for example in situations where a waveguide is being used to couple to a cavity with flat sidewalls. Flat sidewalls provide an increased interaction length when coupled to a straight waveguide. This enables more light to enter the cavity. Changing the cavity shape has also been shown to cause changes in the free spectral range which may be beneficial for certain applications, such as tracking a specific resonance mode. For example, microtoroids have fewer transverse and radial modes than microspheres [7]. It has recently been shown that microdisk resonances can also be finely tuned using chemical etching to precisely control the resonance frequency [62]. This process cannot be done on STIMs.

One tradeoff of different cavity configurations compared to silica microspheres and toroids is that these resonators typically have lower *Q* values, making silica spheres and toroids preferable for low concentration biological and chemical sensing. An exception is the CaF_2_ crystalline cavity (shape shown in Figure 3g), which exhibits *Q* > 10^10^, but is not easily multiplexed and is not as easy to functionalize as silica [63]. CaF_2_ resonators are diamond-cut out of a crystalline blank and hand polished using diamond abrasives.

In terms of cavity material, sapphire resonator biosensors have been used to detect DNA [64]. Liquid droplet resonators have been used to measure concentrations of oil. These resonators behave as both the resonator and the sample. In these resonators, evaporation can speed up reactions, enabling lower limits of detection, but limiting experiment times [65]. Diamond resonators are also potentially attractive biosensors. These resonators are grown using microwave plasma assisted chemical vapor deposition. Biomolecules may be attached to diamond surfaces [66] and diamond resonators have high Q-values in excess of 10^7^. Diamond resonators have the advantage of being extremely stable to refractive index changes due to temperature fluctuations [53].

Other variations on cavity biosensing platforms include using a sol-gel coating as a means to incorporate a gain medium into the optical system to make a narrow linewidth laser which can be used for sensing purposes [67]. This is a cost-effective and customizable alternative to doping glass with a rare earth element. Embedding quantum dots within a microsphere has also been shown to enable resonant mode excitation from the far-field thus enabling remote sensing [68].

## 4. Principles behind Whispering Gallery Mode Optical Resonator Based Sensing

### 4.1. Light Coupling

**Figure 4:**
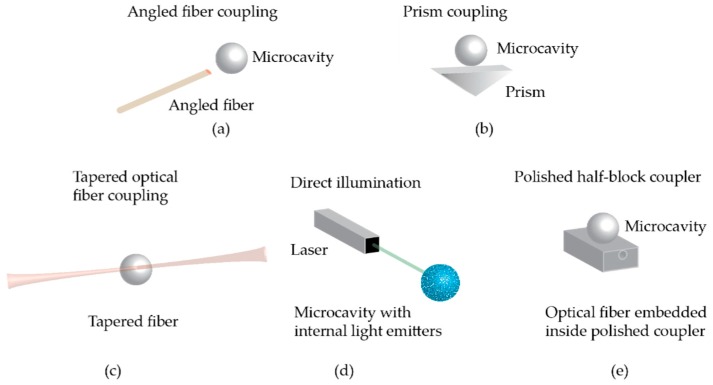
Schematic of different ways to couple light into a symmetric optical microcavity: (**a**) Angled fiber coupling; (**b**) Prism coupling. While robust, prism coupling is difficult to achieve for on-chip devices; (**c**) Tapered optical fiber coupling. The waist of the fiber has been thinned to typically ~500–2000 nm in diameter; (**d**) Direct illumination in the case where the resonator has internal light emitters such as quantum dots. This enables remote sensing; and (**e**) Polished half-block coupler. In a half block coupling system, an optical fiber is embedded in a transparent block and mechanically polished until part of the fiber cladding is removed such that light evanesces out.

Light can be efficiently coupled into symmetrical optical cavities using a phase-matched evanescent field (Figure 4). Coupling is often achieved via a tapered optical fiber positioned in the evanescent zone of the resonator [69] which allows for 99.8% coupling efficiency. This method of coupling light into these optical cavities was first developed in 1997 [70] and is limited by material absorption, scattering, and bending losses due to any physical stress on the fiber. Other forms of light coupling are prism coupling [71], direct illumination in the case where there are light sources within the resonator [26], half-block couplers [72], and the use of angle-polished fibers [73]. These methods have the advantage of being more robust, but are less efficient. Angle-polished fibers have a demonstrated coupling efficiency of >60% [73] while prisms have been experimentally shown to have at most an efficiency of ~80% [71,74]. Half-block couplers have been shown to be more efficient compared to a tapered optical fiber at exciting the zeroth order mode of a microsphere resonator [72].

### 4.2. Whispering Gallery Modes in a Sphere

In 1908, Gustav Mie’s solutions to Maxwell’s equations predicted that the electromagnetic field within a dielectric sphere would exhibit resonance peaks that depend on the size of the particle. From Mie theory, these resonances are affected by cavity size, relative index of refraction with respect to the surrounding medium, and properties of the incoming light such as polarization, wavelength, angle, and beam profile. The existence of resonances within a sphere were also explained by Nussenzveig and Johnson using an effective-potential approach [75,76].

Dielectric spheres have experimentally demonstrated resonant modes with *Q* factors as high as 10^10^, which is close to the theoretical limit caused by absorption of light by the resonator material [32]. This is eleven orders of magnitude lower than the *Q* predicted by Lorenz–Mie theory in the absence of material absorption. In the near-infrared, a *Q*-factor as high as 8 × 10^9^ in air has been reported [77]. From [32], the material limited *Q* ($Q_{mat}$) in air for a silica microsphere at a wavelength (λ) of 633 nm is given as follows, where n is the index of refraction of silica, and α is the absorption coefficient for silica:

(2) $$Q_{mat}=\frac{2\pi n}{\alpha \lambda }=\;0.9\times 10^{10}.$$

Solving Maxwell’s equations inside a sphere show that the resonant modes are leaky modes and the electric field of these modes outside the sphere decays radially away from the surface of the sphere as 1/r, where r is the distance from the center of the sphere. This can be approximated over a short distance as an exponential function corresponding to an evanescent field [33]. In frequency space, the distribution of light near a MDR follows a Lorentzian shape. The electromagnetic field distribution within a cylindrical cavity may be similarly obtained. Although spheres have high *Q* values, they have a dense mode spectrum, e.g., free spectral range ~0.23 nm for a 400 micron diameter sphere at ~1340 nm [78]. This complicates sensing experiments, for example, in cases where a laser is locked to a particular frequency. The free spectral range between modes may be increased, for example, by squashing the sphere into an oblate spheroid [79].

### 4.3. Whispering Gallery Modes in a Toroid

Microtoroids have an advantage over microspheres for biological and chemical sensing in that they are amenable to wafer-based fabrication, which can provide parallel fabrication and easier fluidic handling. Like microspheres, microtoroid optical resonators have ultra-high-*Q* factors (>10^8^). Although analytic solutions for the modes within a toroid do not exist, these modes have been extensively simulated using finite element analysis [80]. Microtoroids have a simpler mode spectrum than a sphere, whose mode spectrum is sensitive to variations in sphere eccentricity [81]. Because a perfect sphere cannot be achieved, the degeneracy in the azimuthal modes leads to splitting, which results in a more complicated mode structure. In contrast, a toroid, due to its confining geometry, has a “cleaner” mode spectrum. This makes it easier for multiple toroids with unique resonances to potentially be coupled to the same optical fiber without the resonances overlapping, allowing multiplexing with the use of only one photodetector.

Microtoroids, although having a ring shape, are different from microring resonators. Unlike the microring, the microtoroid is on a pedestal, meaning that the evanescent field emanating from the microtoroid will not be scattered due to interaction with the underlying substrate. This, combined with a heat reflow process to eliminate lithographic blemishes and other surface imperfections, enables a significantly higher *Q* and, in turn, higher sensitivity to lower concentrations of analyte to be measured. In addition, the fact that the sensing region of the microtoroid is off the substrate reduces the influence of the substrate on the local sensing environment. Microrings and disks have a similar modal structure to a torus, although the frequency spacing between different radial and/or transverse modes can differ between microrings, microdisks, and microtoroids.

### 4.4. Cavity Perturbation Due to Particle Binding

When a particle adsorbs to the surface of an optical resonator, the microcavity is perturbed and the resonance frequency changes. These frequency changes can be sensitively measured. The relationship between the particle diameter and the change in resonance frequency observed upon binding was developed in 1945 and is known as the Bethe–Schwinger cavity perturbation formula [33,82]:

(3) $$\frac{\omega -\omega_{0}}{\omega }\approx \frac{\int_{\Delta V}\left[E_{0}^{*}\varepsilon_{0}\Delta \varepsilon \left(r\right)E_{0}+H_{0}^{*}\mu_{0}\Delta \mu \left(r\right)H_{0}\right]dV}{\int_{V}\left[\varepsilon_{0}\varepsilon \left(r\right)E_{0}^{*}\cdot E_{0}+\mu_{0}\mu \left(r\right)H_{0}^{*}\cdot H_{0}\right]dV},$$

where ε(r) and μ(r) are the local relative permittivity and permeability of material at position r, ω is the resonance frequency of the cavity, $\omega -\omega_{0}$ is the change in resonance frequency of the cavity after the particle binds, $E_{0}$ and $H_{0}$ are the electric and magnetic fields, respectively, in the absence of a particle, V is the total integration volume covering the full mode field, and ΔV is the volume of the particle. While Δε(r) is in general be a 3 × 3 tensor, it can often be reduced to a scalar for isotropic materials. Assuming that: (1) the magnetic permeabilities of all the materials are equal (μ=1), then Δμ=0; (2) the electric fields are approximately constant across the small size of the particle; (3) there are equal amounts of energy in the electric and magnetic fields; and (4) we can write $E_{0}^{*}E_{0}=\;{\left|E_{0}\right|}^{2}$, Equation (3) may be reduced to:

(4) $$\frac{\Delta \omega }{\omega }\approx \frac{\varepsilon_{0}\Delta \varepsilon {\left|E_{0}(r_{particle})\right|}^{2}\Delta V}{2\int_{V}\left[\varepsilon_{0}\varepsilon \left(r\right){\left|E_{0}\right|}^{2}\right]dV}$$

The numerator refers to parameters involving the particle, and the denominator refers to the cavity mode volume. Via the Clausius–Mossotti relation,

(5) $$\alpha =3\varepsilon_{0}V_{0}\frac{\Delta \varepsilon }{3\varepsilon_{medium\;}+\Delta \varepsilon },$$

the frequency shift can also be expressed in terms of the polarizability of the particle. From Equation (4), the shift is dependent on the mode volume of light within the cavity, the volume of the particle being detected, and the polarizability of the particle.

### 4.5. Other Detection Configurations, Mechanisms, and Improvements

**Figure 5:**
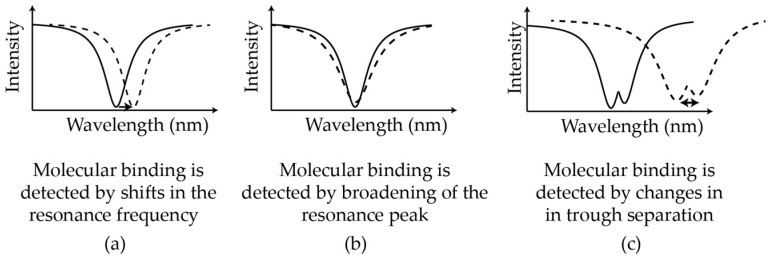
Summary of different sensing modalities: (**a**) particle binding events are detected by measuring shifts in the resonance frequency of the optical cavity; (**b**) binding events are detected by broadening of the resonance peak, reflecting a drop in the quality factor of the cavity due to scattering; and (**c**) particle binding is sensed by measuring changes in trough separation of a resonance doublet. These doublets are created by scattering caused, for example, by the deposition of a nanoparticle on the resonator surface.

In addition to measuring resonance frequency shifts of these devices as particles bind, there are several other detection mechanisms (Figure 5). These include split-frequency sensing [83,84,85,86], resonance broadening [87], and optomechanical oscillations [88,89]. Split frequency sensing is when resonance doublets occur due to backscattering within the cavity from, for example, a manufacturing defect. Tracking the distance between the two resonance peaks as particles bind provides a self-referential sensing scheme that is robust to environmental noise. Microtoroid split-mode Raman lasers have also been used to detect sodium chloride particles of radius 10 nm in air [90] and polystyrene particles of radius 20 nm in water [91]. These devices use the intrinsic Raman gain of silica thus eliminating the need for a dopant. In optomechanical sensing, radiation pressure from the light within the cavity results in mechanical oscillations of the cavity. The degree of oscillation is affected by particle binding. Q-broadening simply refers to degradation of the cavity-Q due to scattering as particles bind. A recent development known as dual frequency comb spectroscopy, involves mixing the pulse trains from two disk resonators to generate an absorption spectrum which may provide a means to identify a particular chemical species [92].

### 4.6. Surface Functionalization

Surface functionalization becomes important when performing biological and chemical detection experiments in complex solutions such as bodily fluids or wastewater. As the most commonly used resonators are made of glass, functionalizing their surface for biological detection is done by binding capture agents such as antibodies, complementary DNA (cDNA), and aptamers using silane linkers. One preferable means of functionalizing WGM sensors is to vapor deposit linker molecules such as 3-Aminopropyltrimethoxysilane (APTES). This provides a uniform coating which allows high-Q values to be maintained. Biological molecules can then be bound to the amine group on APTES molecules using EDC-NHS chemistry. Thin polymer layers have also been shown as an alternative to APTES [93].

**Figure 6:**
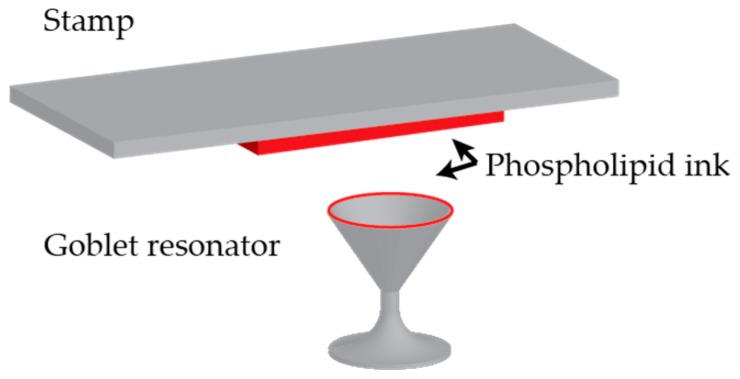
Goblet resonators are functionalized with a phospholipid “ink” using a stamping procedure which allows for each resonator in to be functionalized with a different capture agent in a parallel fashion.

For multiplexed assays, goblet resonators (Figure 6) have been functionalized using a stamping procedure which allows each resonator to be simultaneously functionalized with a different capture agent and eliminates the need for bulk incubation, thus decreasing the amount surface functionalization agents needed [45].

## 5. Recent Developments in Biological and Chemical Sensing

Recently, two main technological advances have been used to further push the sensitivity of these detectors. These include plasmonic enhancement, which amplifies the detected signal, and frequency-locking, which suppress background noise.

### 5.1. Plasmonic Enhancement

**Figure 7:**
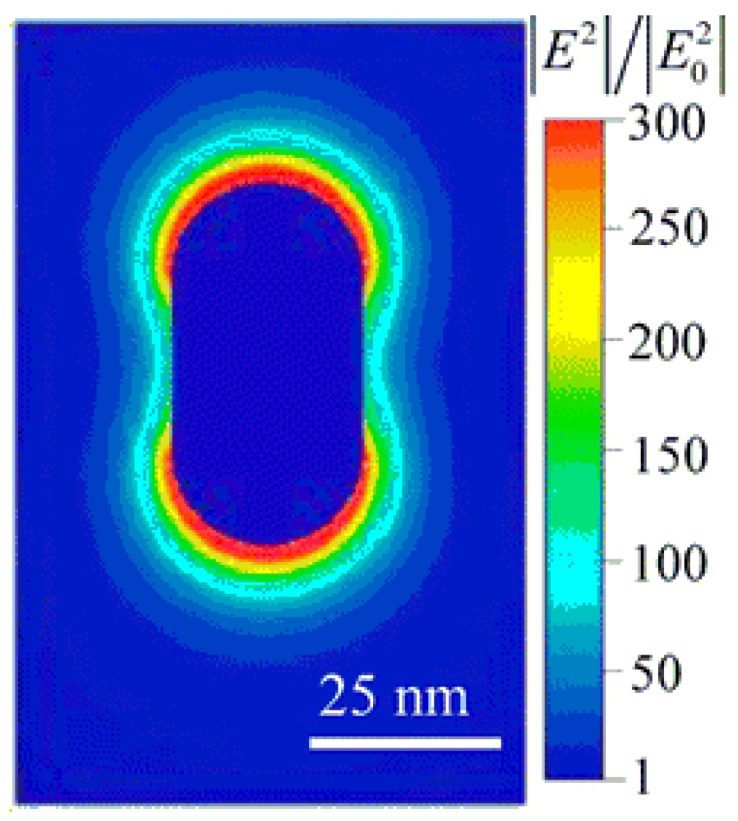
Finite element simulation of the intensity enhancement due to localized surface plasmon resonance of a gold nanorod. *E*_0_ represents the field from the incident light and *E* represents the local field in the vicinity of the nanorod. Reprinted by permission from Macmillan Publishers Ltd.: Nature Nanotechnology [94]), copyright (2012).

Plasmonic enhancement stems from the coupling between a plasmonic resonance and the WGM resonance. On their own, resonant metal nanoparticles have been used for biological sensing [10,94] and have advantages over fluorescent tags and quantum dots through not bleaching or blinking; however, the resonance linewidth of these particles is often much wider than the spectral shift anticipated from refractive index changes caused by a molecular binding event, which makes binding events exceedingly difficult to detect. In other words, plasmonic particles have low *Q*. When these particles are coupled to a WGM resonator, the resonance linewidth of the combined system is much narrower [18], enabling smaller spectral shifts to be measured than for the single nanoparticle alone. On the other hand, the benefit of the coupled system over a WGM resonator by itself is that the nanoparticle serves to enhance the interaction between the light and analyte particles. A larger shift in resonance is observed for the same analyte particle binding to a hybrid system instead of a bare WGM system. Such hybrid WGM resonators have been used to detect single BSA (~14 nm) molecules [18] and atomic ions [95]. Recent work using a gold nanorod on a microsphere demonstrated ~1000× signal enhancement (Figure 7) [15,96] over a non-hybrid WGM resonator. Although this is a means to increase the SNR of the sensing system, metallic nanoparticles have inconsistent signal amplification due to inherent non-uniformities in manufacturing and, as mentioned previously in the Introduction, significantly decrease the capture area of the sensor.

### 5.2. Frequency-Locking

**Figure 8:**
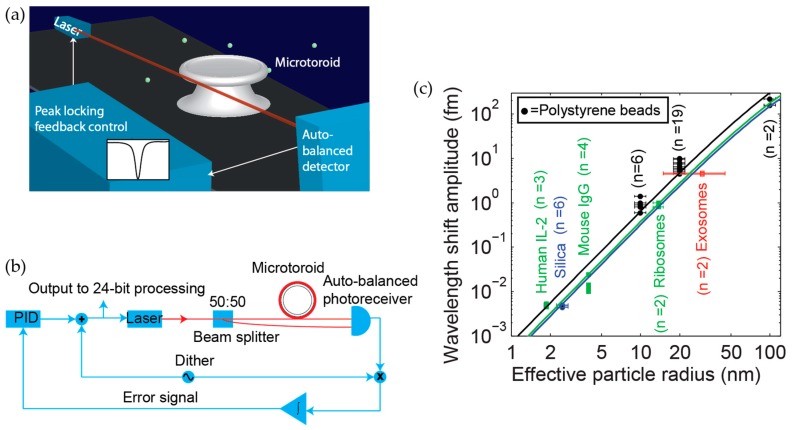
Overview of FLOWER and summary of particle detection data (**a**) FLOWER schematic. Frequency locking in combination with auto-balanced detection and data processing techniques have been used to improve the signal-to-noise ratio of microtoroid optical resonators to the extent that single macromolecule detection is possible. Reprinted (adapted) with permission from [16]. Copyright 2015 American Chemical Society; (**b**) Block diagram of FLOWER. Reproduced from [1]; and (**c**) Summary of particle detection data acquired using FLOWER. The different colored solid lines are theoretical predictions corresponding to different particle dielectric constants. Reproduced from [1].

We have recently developed a technique known as a frequency locked whispering evanescent resonator (FLOWER) (Figure 8a), which uses frequency-locking in combination with balanced detection (Figure 8b) and nonlinear post-processing techniques to significantly improve the signal-to-noise (SNR) ratio of silica microtoroid optical resonators to the extent that single 14.5 kDa molecules can be detected on the surface of a bare (non-plasmonic hybrid) toroid [1,17]. Frequency locking had previously been used as a means to sense 39 nm × 10 nm gold nanorods [97], but not biological molecules. The toroids used in these experiments were ~90 microns in diameter. A summary of various particles detected using FLOWER is shown in Figure 8c.

**Figure 9:**
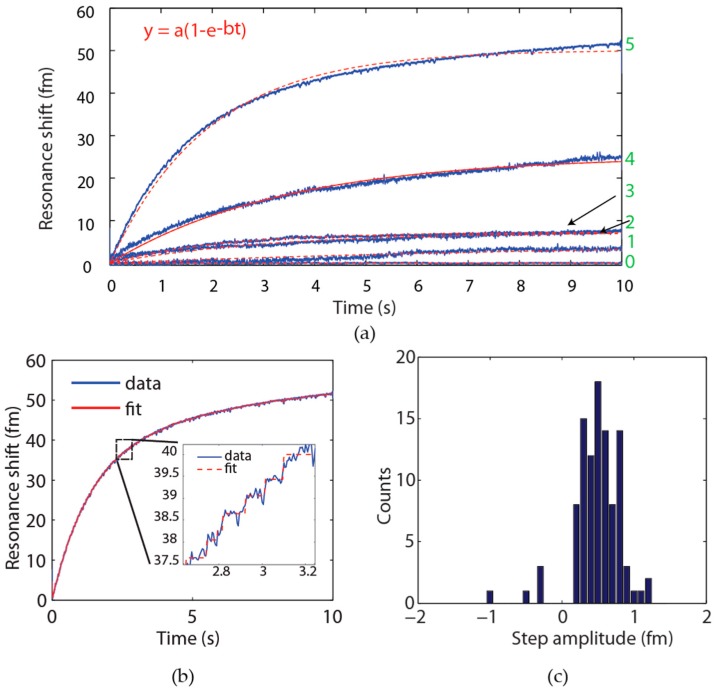
Exosome detection data obtained using FLOWER (**a**) Exosome binding curves. Mice were implanted with human Burkitt’s lymphoma tumor cells, and each week blood serum samples were taken and later analyzed all together using FLOWER. The curves shown here are from a single mouse. For each week we see an increase in the response from the sensor corresponding to increasing exosome levels. Weeks are indicated in green. No significant signal was obtained from Week 0. The data traces are fit with a simple exponential (dashed red line) corresponding to first-order kinetics. (**b**) Zoom-in of Week 5 and corresponding step-fit (red) and (**c**) histogram of step heights. Individual steps corresponding to the binding of individual exosomes may be seen. Negative step amplitudes represent unbinding events. Reprinted (adapted) with permission from [16]. Copyright 2015 American Chemical Society.

As one example of our ability to selectively detect biomolecules in complex media, we have used FLOWER to successfully sense low concentrations of exosome (~40 nm nanovesicle) cancer biomarkers in mouse serum (Figure 9) [16]. A sensor that can detect a minute quantity of exosomes can potentially determine the existence and progression of a tumor based upon the circulation of blood without the need to find and access the tumor. Recently, a similar frequency-locking scheme was combined with absorption spectroscopy to measure the absorption spectra of gold nanorods in air on a microtoroid [98].

It has also been discovered that optical trapping forces within a microcavity enhance particle transport to the resonator, thus facilitating particle detection [99]. Analyte binding can also be enhanced by adding more salt to the binding solution, as this decreases the Debye length of the solution and screens any electrostatic repulsion acting on particles with a similar chemical surface charge as the sensor.

### 5.3. Recent Developments in Chemical Sensing

WGM sensors have been used to detect a variety of chemical species, including gases and explosives. For chemical sensing, traditional antibody-based biosensing approaches cannot be used. Instead, a typical approach is to coat the resonator with a chemically selective polymer [100]. As the polymer absorbs chemicals, it swells, increasing the diameter of the resonator. Specific polymer responses can be correlated to specific chemicals. In one example, chemoselective sorbent (HCSFA2) coatings on microring resonators were used to detect vapors of acetone, dimethyl methylphosphonate (DMMP), and nitrobenzene [101]. These coatings were deposited on the resonator using a microcapillary tip. The detection limit of microcylinder resonators for chemical vapor sensing using chemoselective polymer coatings has been estimated to be 0.1 ppm [102]. An ethylene/propylene polymer was used by microrings to detect o-xylene in solution [103].

Besides polymer coatings, other ways of imparting chemical selectivity to WGM resonators have been used. Capillary electrophoresis has been combined with microring resonators to separate and detect sodium, lithium, and potassium cations in a mixture [104]. Zeolite nanoporous film coatings have been proposed as a way to selectively detect chemical vapors [105]. These films have been shown to be able to detect varying concentrations of isopropanol, benzene, and acetone vapor [106]. Selectivity is based on the zeolite pore size. In another experiment, ammonia was detected by microrings using a silica film sensing layer deposited using atomic layer deposition [107]. In these experiments, Brønsted acid sites in the film favor the binding of ammonia, which is a base. TNT has also been selectivity detected using microring resonators electrosprayed with triphenylene-ketal receptor molecules [108].

### 5.4. Sensitivity and Speed Comparison among Widely Used WGM Optical Geometries

In terms of reported sensitivity for protein detection, FLOWER, which is based on silica microtoroid technology (see Section 5.2), has demonstrated single macromolecule detection at attomolar concentrations [1,17]. Hybrid WGM resonators (Section 5.1) have demonstrated femtomolar detection of BSA [18] and silica microrings can routinely achieve picomolar detection of proteins such as interleukin-2 [109].

**Figure 10:**
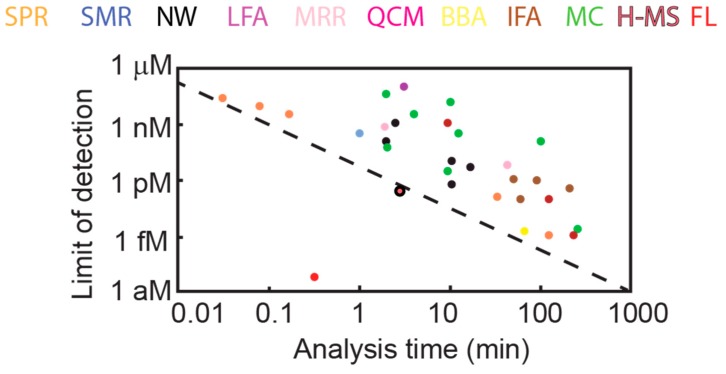
A comparison of biosensing techniques for protein sensing. SPR = Surface Plasmon Resonance; SMR = Suspended Microchannel Resonators; NW = Nanowires; LFA = Lateral Flow Assay; MRR = Microring Resonator; QCM = Quartz Crystal Microbalance; BBA = BioBarcode Assay; IFA = Immunofluorescence Assay; MC = Microcantilever; H-MS = Hybrid-Microsphere [18]; FL = FLOWER. The dashed line represents the present state of the art, not including recent advances in WGM sensing. For cases where the limit of detection is determined by analyte mass, a molecular weight of 34 kDa is assumed (Partially adapted from [11]).

Due to the label-free nature of WGM sensors, the time in which results may be obtained with WGM sensors is significantly less than for labeled assays such as ELISA, which takes hours and requires multiple processing steps. As a point of comparison, microrings can obtain results in 10–30 min, and FLOWER can obtain results in under 30 s, which is the approximate time it takes for the sensor to reach steady-state after injection. Compared to other nanoscale sensors, WGM sensors, depending on the geometry, can be faster and more sensitive (Figure 10). Figure 10 includes a sampling of commonly used WGM geometries (sphere, toroid, and ring) in comparison to other protein sensing technologies.

## 6. Outlook

A major area for improvement in the near future is in the multiplexing of WGM resonator sensors. Other techniques, such as arrayed imaging reflectometry [110], have proven the importance of robust multiplexed optical sensors. A limiting factor in multiplexing WGM resonators is the lack of a robust way to couple cavities to sources and detectors while maintaining high sensitivity. Once this has been achieved, high-*Q* WGM systems can be made highly multiplexed with multiple viable sensors in one platform. Recently, a robust coupling solution was demonstrated in a thermal sensing platform where the fiber and a toroidal microcavity were both encapsulated in a polymer matrix [111]. Such a platform would not work well in solution, however because the polymer matrix limits the transport of analyte to the surface of the WGM resonator.

Beyond multiplexing, many other improvements can be made to these devices. Improved sensitivity can enable the detection of even smaller molecules of societal interest, such as insulin. We also expect that in the future, these sensors will be made re-usable while still maintaining high cavity-*Q*. For widespread implementation of WGM resonator sensors, further improvements need to be made in cost, speed, portability, and selectivity. With these advances, results can be obtained on-site, instantaneously, with zero false negatives, and at a widely accessible cost. One of the limiting factors in cost is the efficient mass production of high-*Q* devices. Microtoroids were recently batch-fabricated using an oven, which is a more parallel than carbon dioxide laser reflow. The oven-reflow process created microtoroids with *Q*-values that were mid-10^6^ [112]. Finally, another barrier to widespread implementation has been lack of characterization of these devices with a known reference standard such as Biacore surface plasmon resonance or ELISA. As the field expands, we anticipate that such experiments will be performed.

We anticipate that the application of WGM sensors will enable fundamental studies of single protein-protein and receptor-ligand interactions. We further expect these sensors to find great use in medical diagnostics, environmental monitoring, chemical threat sensing, and food and water quality applications. In terms of medical diagnostics, we expect these sensors will be used in personalized medicine, where these sensors could perhaps be used to evaluate, for example, the efficacy of a particular cancer treatment for an individual. In the future, it is likely that further developments in the reliability and sensitivity of these sensors for gas detection will be achieved [108,113]. This would enable applications such as monitoring ammonia from spoiled meat. Eventually, we predict that we will be able to use many of these sensors in one device to detect multiple analytes from a distance, similar to a “tricorder”, or even placed within the body for high sensitivity in vivo biological and chemical sensing experiments.
